# 骨髓增生异常综合征患者铁代谢评估影响因素的回顾性研究

**DOI:** 10.3760/cma.j.issn.0253-2727.2022.04.005

**Published:** 2022-04

**Authors:** 耀 张, 超 肖, 菁 李, 路茜 宋, 幼山 赵, 俊功 赵, 春康 常

**Affiliations:** 上海市第六人民医院，上海 200233 Sixth People's Hospital Affiliated to Shanghai Jiao Tong University, Shanghai 200233, China

**Keywords:** 骨髓增生异常综合征, 磁共振成像, 能谱CT, 铁过载, 去铁治疗, Myelodysplastic syndrome, MRI, DECT, Iron overload, Iron chelation therapy

## Abstract

**目的:**

分析骨髓增生异常综合征（MDS）患者铁代谢评估的影响因素。

**方法:**

181例MDS患者接受磁共振成像（MRI）和（或）能谱CT（DECT）对肝脏和心脏铁浓度的检测，其中41例患者在2次检查期间接受了规律铁螯合治疗（ICT）。同步检测调整铁蛋白（ASF）、红细胞生成素（EPO）、心功能、肝转氨酶、肝炎抗体、外周血T细胞极化等指标，并收集患者是否合并骨髓纤维化、脾大、环孢素A使用等信息进行比较分析。

**结果:**

MRI组、DECT组肝铁浓度均与ASF呈正相关（*r*分别为0.512、0.606，*P*值均<0.001），MRI组心铁浓度与ASF仅呈弱相关（*r*＝0.303，*P*<0.001），而DECT组心铁浓度与ASF无明显相关性（*r*＝0.231，*P*＝0.053）。输血依赖显著影响患者肝和心铁浓度［MRI组：LIC：（28.370±10.706）mg/g对（7.593±3.508）mg/g，*t*＝24.30，*P*<0.001；MIC：1.81对0.95，*z*＝2.625，*P*<0.05，DECT组：肝VIC：（4.269±1.258）g/L对（1.078±0.383）g/L，*t*＝23.14，*P*<0.001：心VIC：1.69对0.68，*z*＝3.142，*P*<0.05］。重度以上铁过载组患者EPO浓度明显高于轻中度铁过载组及正常组（*P*值均<0.001）；MDS伴环状铁粒幼红细胞（MDS-RS）患者与和其他MDS低危组患者相比，肝铁浓度明显增高［DECT组：3.80（1.97,5.51）g/L对1.66（0.67,2.94）g/L，*P*＝0.004；MRI组：13.7（8.1,29.1）mg/g对11.6（7.1,21.1）mg/g，*P*＝0.032］。而年龄、骨髓纤维化、脾大、T细胞极化、环孢素A的使用、肝转氨酶、肝炎抗体阳性等因素对铁代谢无明显影响。

**结论:**

MDS患者肝铁浓度与ASF呈正相关，心铁浓度与ASF无明显相关性。输血依赖、EPO浓度、合并RS是铁代谢的影响因素。

## Abstract (en)

**Objective:**

To analyze the influencing factors of iron metabolism assessment in patients with myelodysplastic syndrome.

**Methods:**

MRI and/or DECT were used to detect liver and cardiac iron content in 181 patients with MDS, among whom, 41 received regular iron chelation therapy during two examinations. The adjusted ferritin（ASF）, erythropoietin（EPO）, cardiac function, liver transaminase, hepatitis antibody, and peripheral blood T cell polarization were detected and the results of myelofibrosis, splenomegaly, and cyclosporine were collected and comparative analyzed in patients.

**Results:**

We observed a positive correlation between liver iron concentration and ASF both in the MRI group and DECT groups（*r*＝0.512 and 0.606, respectively, *P*<0.001）, only a weak correlation between the heart iron concentration and ASF in the MRI group（*r*＝0.303, *P*<0.001）, and no significant correlation between cardiac iron concentration and ASF in the DECT group（*r*＝0.231, *P*＝0.053）. Moreover, transfusion dependence in liver and cardiac [MRI group was significantly associated with the concentration of iron in: LIC:（28.370±10.706）mg/g *vs*（7.593±3.508）mg/g, *t*＝24.30, *P*<0.001; MIC: 1.81 *vs* 0.95, *z*＝2.625, *P*<0.05; DECT group: liver VIC:（4.269±1.258）g/L *vs*（1.078±0.383）g/L, *t*＝23.14, *P*<0.001: cardiac VIC: 1.69 *vs* 0.68, *z*＝3.142, *P*<0.05]. The concentration of EPO in the severe iron overload group was significantly higher than that in the mild to moderate iron overload group and normal group（*P*<0.001）. Compared to the low-risk MDS group, the liver iron concentration in patients with MDS with cyclic sideroblasts（MDS-RS）was significantly elevated [DECT group: 3.80（1.97, 5.51）g/L *vs* 1.66（0.67, 2.94）g/L, *P*＝0.004; MRI group: 13.7（8.1,29.1）mg/g *vs* 11.6（7.1,21.1）mg/g, *P*＝0.032]. Factors including age, bone marrow fibrosis, splenomegaly, T cell polarization, use of cyclosporine A, liver aminotransferase, and hepatitis antibody positive had no obvious effect on iron metabolism.

**Conclusion:**

There was a positive correlation between liver iron concentration and ASF in patients with MDS, whereas there was no significant correlation between cardiac iron concentration and ASF. Iron metabolism was affected by transfusion dependence, EPO concentration, and RS.

铁过载影响血液病患者的自然病程及生活质量，过量的铁广泛沉积在肝脏、心脏和其他器官，可导致结构损伤和功能障碍，尤其是在合并肝炎和贫血心肌病的患者中[1]–[2]。70％以上的铁沉积在肝脏，肝铁浓度可作为评价体内铁浓度的指标。目前临床主要使用磁共振成像（MRI）和能谱CT（DECT）进行肝铁浓度评估。DECT对肝铁的诊断性能和MRI相当[3]，根据三物质分离原理，重建出的虚拟铁浓度（VIC）成像能够剔除脂肪干扰因素[4]。在骨髓增生异常综合征（MDS）患者中，输血不是铁过载的唯一原因，无效造血[5]、铁代谢异常也会导致铁过载。淋巴细胞对于铁调素的表达至关重要，当T淋巴细胞活化后，铁调素mRNA表达水平升高[6]，CD4^+^高表达和Th1/Th2高的患者铁调素水平显著高于正常组[7]。环孢素A（CsA）可改变T细胞的功能，减少炎症因子的产生[8]。合并骨髓纤维化时骨髓造血微环境的破坏会加重造血功能衰竭，合并脾脏髓外造血及脾功能亢进可滞留和破坏循环中的红细胞[9]。本文通过分析MDS患者铁过载的影响因素，为更准确评估铁浓度及去铁疗效提供参考。

## 病例与方法

1. 病例资料：2014年9月至2021年5月共181例MDS患者纳入本研究。按照《骨髓增生异常综合征中国诊断与治疗指南（2019年版）》[10]，MDS伴原始细胞增多（MDS-EB）38例，MDS伴环状铁粒幼红细胞（MDS-RS）31例，MDS伴多系血细胞发育异常（MDS-MLD）110例，MDS伴单系血细胞发育异常（MDS-SLD）2例。41例预期生存期≥1年、输血总量超过80 U（200 ml 全血分离的红细胞为1 U RBC）、调整铁蛋白（ASF）≥1 000 µg/L持续2个月以上、输注依赖[11]的患者接受铁螯合治疗（ICT）：甲磺酸去铁胺20～60 mg·kg^−1^·d^−1^持续皮下泵入12 h或地拉罗司30 mg·kg^−1^·d^−1^口服，14 d为1个疗程[12]。全部患者均治疗4个疗程以上，其中32例完成8个疗程及以上，24例完成10个疗程及以上。患者均接受肝脏和心脏铁浓度检测，其中23例患者同时行MRI和DECT检测，根据我们前期研究经验，将ASF<2 500 µg/L的9例患者归入MRI组，ASF≥ 2 500 µg/L的14例患者归入DECT组进行后续分析。铁过载程度定义：参照文献[13]，DECT组中肝VIC<1.34 g/L为正常组，（1.34～<1.85）g/L为轻度铁过载组，（1.85～<2.69）g/L为中度铁过载组，（2.69～<4.03）g/L为重度铁过载组，≥4.03 g/L为极重度铁过载组；参照文献[14]定义，MRI组中肝铁浓度（LIC）<1.34 g/L为正常组，（1.34～<1.85）g/L为轻度铁过载组，（1.85～<2.69）g/L为中度铁过载组，（2.69～<4.03）g/L为重度铁过载组，≥4.03 g/L为极重度铁过载组。

2. MRI及DECT检测肝脏和心脏铁浓度：采用德国 Siemens公司 Verio 3.0T磁共振成像系统，由熟练的操作者完成肝脏、心脏影像采集。使用2×192层SOMATOM Force DECT扫描仪（德国西门子公司）完成DECT检测。具体操作参照文献[13]。参照文献[15]，通过公式0.202+25.4/T2*完成从T2*到mg/g的转换。

3. 其他相关指标检测：放射免疫法测定SF、B型利钠肽原（proBNP）和EPO。亚铁氮比色法测定血清铁和总铁结合力。丙酮酸氧化酶法测定肝转氨酶等。免疫比浊法检测C反应蛋白（CRP）。ASF可以减少炎症对铁蛋白的影响。CRP>10 mg/L时ASF＝SF/ln CRP，CRP≤10 mg/L时ASF＝SF[16]。流式细胞术检测外周血T细胞极化，计算出Thl/Th2、Tcl/Tc2的比值。B超检测脾脏大小。心脏多普勒彩色超声测得左心室射血分数（LVEF）。嗜银染色检测骨髓网硬蛋白增生程度：++～+++为合并纤维化。

4. 统计学处理：采用SPSS 17.0进行统计分析。正态分布数据以“均数±标准差”表示，两组数据比较采用独立样本*t*检验，去铁前后数据采用配对*t*检验。非正态分布的数据以中位数表示，两组间比较采用Mann-Whitney *U*检验，两组间治疗前后比较采用关联Wilcoxon检验，相关性分析采用Spearman分析；分类变量采用*χ*^2^检验或Fisher精确概率法。*P*<0.05为差异有统计学意义。多重比较对*P*值进行bonferroni校正。

## 结果

1. 一般资料：181例患者中，男104例，女77例。中位年龄55（21～82）岁。147例患者有输血史，近1年中位输血量为18（4～62）U。其中91例患者合并输血依赖。38例患者接受CsA治疗，其中26例患者6个月后获得血液学缓解。有35例患者合并骨髓纤维化，32例患者合并脾大。DECT组71例患者中肝铁正常19例，轻度至中度铁过载7例，重度铁过载24例，极重度铁过载21例。DECT组31例未输血患者中，肝铁正常28例，轻度至中度铁过载2例，重度铁过载1例。MRI组110例患者中肝铁正常4例，轻度至中度铁过载49例，重度铁过载31例，极重度铁过载26例。MRI组25例未输血患者中，轻度至中度铁过载15例，重度铁过载4例，极重度铁过载2例。

2. ASF与肝脏、心脏铁浓度间的相关性：患者肝铁、心铁浓度之间存在明显的不一致性（图1），不能相互替代。分别观察肝脏、心脏铁浓度与ASF间的关系，结果显示，MRI组、DECT组肝铁浓度均与ASF呈正相关（*r*分别为0.512、0.606，*P*值均<0.001）（图2A、2B）；MRI组心铁浓度（MIC）与ASF仅呈弱相关（图2C），而DECT组心VIC与ASF无明显相关性（图2D）。

**图1:**
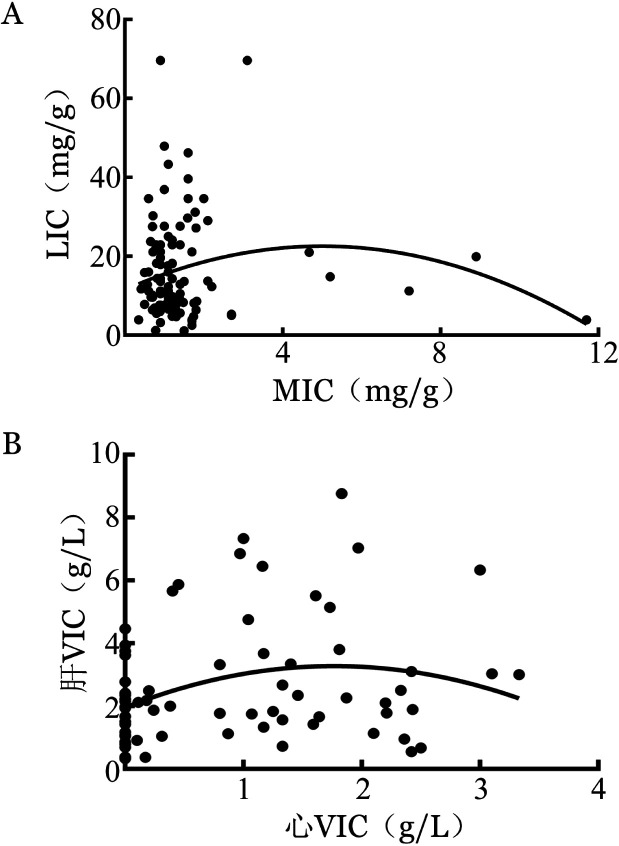
181例骨髓增生异常综合征患者MRI（A）和能谱CT（B）中肝铁、心铁浓度一致性评价 LIC：MRI检测肝铁浓度；MIC：MRI检测心铁浓度；VIC：虚拟铁浓度

**图2:**
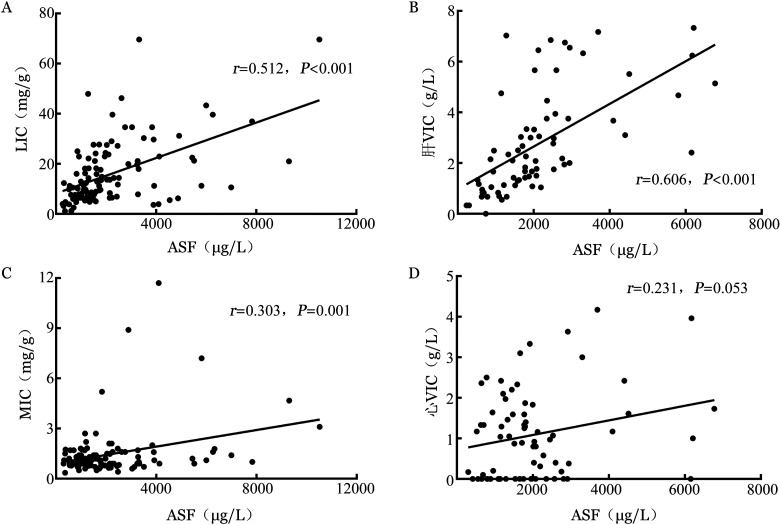
181例骨髓增生异常综合征患者MRI和能谱CT中肝铁、心铁浓度与调整铁蛋白（ASF）的相关性分析 LIC：MRI检测肝铁浓度；MIC：MRI检测心铁浓度；VIC：虚拟铁浓度。A：LIC与ASF；B：肝VIC与ASF；C：MIC与ASF；D：心VIC与ASF

3. 输血依赖对肝铁和心铁浓度的影响及ICT前后的变化：91例患者输血依赖，中位血细胞输注量为52（16～560）U，90例非输血依赖患者中位血细胞输注量为2（0～10）U；输血依赖组肝铁和心铁浓度明显高于非输血依赖组［MRI组：LIC：（28.370±10.706）mg/g对（7.593±3.508）mg/g，*t*＝24.30，*P*<0.001；MIC：1.81对0.95，*z*＝2.625，*P*＝0.013，DECT组：肝VIC：（4.269±1.258）g/L对（1.078±0.383）g/L，*t*＝23.14，*P*<0.001：心VIC：1.69对0.68，*z*＝3.142，*P*＝0.004］。41例接受ICT的患者中，DECT组12例，ICT后ASF、EPO明显下降，但肝VIC和心VIC无明显变化；MRI组29例，ICT后ASF、EPO下降明显，LIC也明显下降，但MIC无明显变化（表1）。

**表1:** DECT组和MRI组去铁前后ASF、肝铁、心铁浓度和EPO的变化

组别	例数	DECT（*x±s*）			
		ASF（µg/L）	肝VIC（g/L）	心VIC（g/L）	EPO（U/L）
ICT前	12	2 708.45±1 259.34	3.84±2.53	1.97±2.09	6 415.33±1 970.51
ICT后	12	2 031.54±1 072.71	2.36±1.91	1.05±1.04	3 671.24±1 327.32

统计量		−3.527	−2.875	−1.544	−3.456
*P*值		0.005	0.017	0.154	0.006

组别	例数	MRI［*M*（范围）］			
		ASF（µg/L）	LIC（mg/g）	MIC（mg/g）	EPO（U/L）
ICT前	29	1 736（1 054, 2 450）	13.6（8.11, 23.91）	1.1（0.80, 1.40）	3 221（653, 9 125）
ICT后	29	1 237（889, 1 524.50）	7.8（6.15, 19.59）	1（0.85, 1.35）	1 646（138, 6 702）

统计量		−2.692	−3.719	−0.456	−3.822
*P*值		0.007	0.001	0.648	<0.001

注：ICT：铁螯合治疗；DECT：能谱CT；ASF：调整铁蛋白；VIC：虚拟铁浓度；EPO：促红细胞生成素；LIC：MRI检测肝铁浓度；MIC：MRI检测心铁浓度

4. EPO浓度与肝铁、心铁浓度间的相关性：MRI组1个月内未使用重组EPO治疗的MDS患者共95例，其EPO浓度与LIC呈较弱的正相关，但与MIC无明显相关性（图3A、3B）。重度以上铁过载组EPO的表达明显高于轻中度铁过载组［7 032（2 187，12 551）U/L对1 381（648，3 084）U/L，*z*＝5.327，*P*<0.001］和正常组［7 032（2 187，12 551）U/L对1 265（466，3 345）U/L，*z*＝5.512，*P*<0.001］。轻中度铁过载组与正常组相比差异无统计学意义（*z*＝0.532，*P*＝0.336）。DECT组1个月内未使用重组EPO治疗的MDS患者共52例，其EPO浓度与肝VIC呈正相关，但与心VIC无明显相关性（图3C、3D）。重度以上铁过载组患者EPO浓度明显高于轻中度铁过载组［（6 843.83±2 621.74）U/L对（1 114.44±614.03）U/L，*t*＝9.528，*P*<0.001］和正常组［（6 843.83±2 621.74）U/L对（1 060.84±422.35）U/L，*t*＝8.710，*P*<0.001］。轻中度铁过载组与正常组相比差异无统计学意义（*t*＝0.336，*P*＝0.739）。

**图3:**
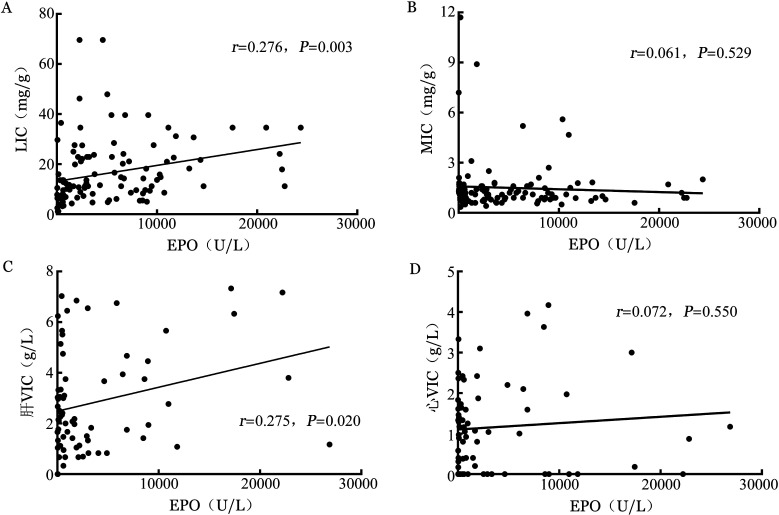
181例骨髓增生异常综合征患者MRI和能谱CT中肝铁、心铁浓度与红细胞生成素（EPO）浓度的相关性分析 LIC：MRI检测肝铁浓度；MIC：MRI检测心铁浓度；VIC：虚拟铁浓度。A：LIC与EPO；B：MIC与EPO；C：肝VIC与EPO；D：心VIC与EPO

5. CsA治疗前后肝铁、心铁浓度的差异性分析：38例患者接受CsA治疗，其中DECT组18例，MRI组20例。12例使用CsA治疗>6个月但仍合并输血依赖的患者，与治疗前比较，其肝VIC（3.29对4.53，*z*＝1.786，*P*＝0.045），LIC（13.22对18.36，*z*＝1.953，*P*＝0.033）均较前加重；心VIC（1.43±1.05对1.62±1.13，*t*＝0.379，*P*>0.05），MIC（1.25对1.38，*z*＝0.4211，*P*>0.05）差异无统计学意义。26例使用CsA治疗有效、脱离输血依赖的患者，与治疗前比较，其肝VIC（3.17±2.64对1.86±1.13，*t*＝3.642，*P*<0.05），LIC（9.58对5.31，*z*＝2.012，*P*<0.05）均较前明显减轻，心VIC（1.64±1.35对1.52±1.23，*t*＝0.2875，*P*>0.05），MIC（1.34对1.41，*z*＝0.336，*P*>0.05）差异无统计学意义。

6. 合并RS、骨髓纤维化、脾大对肝铁、心铁浓度的影响：DECT组15例MDS-RS患者和47例其他MDS低危组患者相比，ASF差异无统计学意义，肝VIC明显增高，心VIC差异无统计学意义；MRI组16例MDS-RS患者和75例其他MDS低危组患者相比，ASF无明显差异，LIC明显增高，MIC无明显差异（表2）。35例合并骨髓纤维化的患者中，28例合并脾大，其中肝铁正常2例，轻中度铁过载14例，重度以上铁过载19例。146例未合并骨髓纤维化的患者中，肝铁正常24例，轻中度铁过载36例，重度以上铁过载86例。两组患者重度以上肝铁沉积的比例为54.3％对58.9％，差异无统计学意义（*P*＝0.619）。

**表2:** MDS-RS患者与其他低危组MDS患者ASF、肝铁和心铁浓度的差异性分析［*M*（范围）］

组别	例数	DECT		
		ASF（µg/L）	肝VIC（g/L）	心VIC（g/L）
MDS-RS组	15	2 027（863, 4 407）	3.80（1.97, 5.51）	1.17（0.00, 1.97）
其他低危MDS组	47	1 652（898, 2 349）	1.66（0.67, 2.94）	0.80（0.00, 1.59）

*z*值		−0.967	−2.917	−1.228
*P*值		0.334	0.004	0.219

组别	例数	MRI		
		ASF（µg/L）	LIC（mg/g）	MIC（mg/g）
MDS-RS组	16	1 279（623, 2 599）	13.7（8.1, 29.1）	1.00（0.81, 1.54）
其他低危MDS组	75	1 475（984, 2 269）	11.6（7.1, 21.1）	1.11（0.84, 1.70）

*z*值		−0.933	−1.978	−0.665
*P*值		0.351	0.032	0.506

注：MDS：骨髓增生异常综合征；MDS-RS：MDS伴环状铁粒幼红细胞；ASF：调整铁蛋白；DECT：能谱CT；VIC：虚拟铁浓度；LIC：MRI检测肝铁浓度；MIC：MRI检测心铁浓度

7. 年龄、肝功能指标及T细胞极化与肝铁浓度的相关性：结果见表3，患者年龄、ALT、总胆红素、r谷氨酰基转移酶（r-GT）、LDH均与肝铁浓度无明显相关性。181例患者中肝炎患者8例（乙型肝炎6例，丙型肝炎2例），2例肝铁正常，4例轻中度铁过载，2例重度以上铁过载；合并肝炎抗体［除外乙型肝炎表面抗体（HBsAb）］阳性115例，其中乙型肝炎核心抗体（HBcAb）阳性65例，乙型肝炎E抗体（HBeAb）阳性41例，HBcAb合并乙型肝炎E抗体（HBeAb）阳性31例，抗戊型肝炎抗体阳性5例，HBcAb合并抗戊型肝炎抗体阳性2例。所有患者的病毒复制均阴性。肝炎抗体（除外HBsAb）阳性的患者中，肝铁正常17例，轻中度铁过载59例，重度以上铁过载39例。非肝炎抗体（包括HBsAb）阳性的66例患者中，肝铁正常14例，轻中度铁过载28例，重度以上铁过载24例。肝炎抗体（除外HBsAb）阳性与否两组患者重度以上肝铁沉积的比例分别为25.2％（39/115）对36.4（24/66），差异无统计学意义（*P*＝0.739），轻中度肝铁沉积的比例分别为51.3％（59/115）对42.4％（28/66），差异均无统计学意义（*P*＝0.332）。62例未使用CsA治疗的患者行外周血T细胞极化检测，结果显示DECT和MRI组患者Th1/Th2、Tc1/Tc2与肝铁浓度均无明显相关性（表3）。

**表3:** 年龄、肝功能指标及T细胞极化与肝铁浓度的相关性分析

参数	年龄	ALT	总胆红素	r-GT	LDH	Th1/Th2	Tc1/Tc2
肝VIC							
*r*值	0.042	0.074	0.183	0.191	0.115	0.140	0.300
*P*值	0.576	0.489	0.158	0.174	0.482	0.445	0.096
LIC							
*r*值	0.086	0.038	0.187	0.153	0.069	0.182	0.195
*P*值	0.287	0.699	0.058	0.126	0.511	0.323	0.147

注：肝VIC：能谱CT组肝脏虚拟铁浓度；LIC：MRI组中肝铁浓度；r-GT：r谷氨酰基转移酶

8. 年龄、心功能指标及T细胞极化与心铁浓度的相关性：年龄、LVEF、E/A比值、肌酸激酶同工酶（CKMB）、B型利钠肽原（proBNP）与心铁浓度均无明显相关性（表4）。6例合并冠心病（其中2例为心肌梗死支架置入术后）患者心铁浓度均正常。62例未使用CsA治疗的患者行外周血T细胞极化检测，结果显示DECT和MRI组患者Th1/Th2、Tc1/Tc2与心铁浓度均无明显相关性（表4）。

**表4:** 年龄、心功能指标及T细胞极化与心铁浓度的相关性分析

参数	年龄	LVEF	E/A比值	CKMB	proBNP	Th1/Th2	Tc1/Tc2
心VIC							
*r*值	0.058	0.041	0.162	0.125	0.436	0.099	0.054
*P*值	0.621	0.684	0.176	0.263	0.187	0.589	0.769
MIC							
*r*值	0.013	0.057	0.204	0.143	0.246	0.122	0.103
*P*值	0.873	0.639	0.092	0.218	0.048	0.253	0.222

注：心VIC：能谱CT组心脏虚拟铁浓度；MIC：MRI组心脏铁浓度；LVEF：左心室射血分数；CKMB：肌酸激酶同工酶；proBNP：B型利钠肽原

## 讨论

本研究结果表明，ASF与肝铁浓度呈正相关。输血依赖组肝铁和心铁浓度均明显高于非输血依赖组。说明ASF和输血依赖是铁代谢的影响因素。输血依赖不仅造成MDS患者的输入铁过多，且输血次数的增多也是导致红细胞无效输注的因素之一[17]。ICT后ASF明显下降，DECT组肝铁降低，心铁无明显变化，MRI组肝铁降低，心铁无明显变化。这些结果表明，ICT后铁在心脏中的减少比肝铁和ASF降低晚，与既往文献报道一致[18]。因此，评估时要考虑去铁后的患者虽然ASF和肝铁已下降，但心铁可能还无明显变化。

本组MDS-RS患者，在ASF无明显差异的情况下，肝铁浓度较其他低危组患者明显增高。说明合并RS可能是铁代谢的影响因素。MDS-RS患者骨髓造血细胞中出现线粒体铁过载，加重线粒体损伤[19]，推测其合并无效造血程度可能更高。MDS合并骨髓纤维化、脾大对肝铁浓度无明显影响，只要无输血依赖，肝铁浓度与其他组患者比较无明显升高。

EPO与ASF和肝铁浓度显著相关，但与心铁浓度无显著相关。重度以上铁过载患者EPO的表达明显高于轻中度铁过载及正常组患者，而轻中度铁过载和正常组患者间EPO表达无明显差异。EPO刺激红细胞分泌的红铁酮能抑制铁调素的表达，增加铁的吸收[20]。推测EPO的增高可能会干扰铁代谢，加重肝铁沉积。ICT后ASF和EPO均出现明显下降，可能去铁治疗后EPO的降低对减少铁负荷也起到积极作用。

本研究显示Th1/Th2、Tc1/Tc2与肝铁和心铁并无明显相关性。Ⅰ型T细胞极化产生更多的TNF-α及炎性因子，体外实验发现TNF-α可促进铁离子内流调控蛋白转铁蛋白结合受体1（TFR1）和二价金属转运蛋白（DMT1）的表达，抑制膜铁转运蛋白（FPN）的表达，从而促进铁离子内流，抑制铁离子外流，造成细胞铁过载加重[21]。但Th1极化也促进铁调素的表达，减少肠道和巨噬细胞向血液中输送铁。CsA的使用虽然可使Th1表达下降[8]，但患者仍合并输血依赖，肝铁不会出现明显降低,反而会随输血量的增加而增高。

肝铁浓度与年龄、LDH、ALT、r-GT、TBL均无明显相关性。在无肝炎病毒复制的MDS患者中，HBcAb和/或HBeAb及/或抗戊型肝炎抗体的阳性表达对肝铁浓度无明显影响。既往文献表明，慢性肝炎早期可明显抑制铁调素的表达，但病毒因子的负作用可随着铁离子的积累而消除，在肝炎抗体阳性但无病毒复制的患者中，病毒因子的负面影响可以消除[22]。合并活动性肝炎（乙型肝炎和丙型肝炎）的MDS患者由于病例太少，目前尚不能得对肝铁浓度影响的结论。

本研究显示心铁浓度与年龄、LDH、LVEF、E/A比值、CKMB均无显著相关性。MRI组MIC与proBNP有明显相关性，而DECT组心VIC与proBNP无明显相关性。心脏和肝脏对铁的吸收和排泄机制不同，铁代谢动力学不同导致心铁的沉积和去除延迟。心铁浓度不能正确评估全身铁过载[23]。DECT组与MRI组结果的不同，可能与低铁浓度下心VIC测值不准有关，但也不排除患者选择偏倚的影响。

我们前期研究表明，DECT在低铁浓度ASF<1 000 µg/L时、MRI在高铁浓度ASF>5 000 µg/L时准确性较低[13]。本研究DECT检测组中ASF<1 000 µg/L的患者共19例，MRI检测组中ASF>5 000 µg/L的患者共13例。这部分患者对肝铁和心铁浓度检测存在不准确性，可能会影响相关数据的分析结果。

综上所述，本研究结果显示ASF、输血依赖、EPO浓度、MDS-RS、近期行ICT治疗可能是铁代谢评估的影响因素。而在低中度铁过载时，EPO对肝铁的影响不大。心铁浓度与肝铁和ASF均无明显相关性，需独立检测。
